# Gene Expression of *GqBursicon* in *Gynaephora qinghaiensis* (Lepidoptera: Lymantriidae) and Its Impact on Wing Expansion

**DOI:** 10.3390/ijms27093929

**Published:** 2026-04-28

**Authors:** Guixiang Kou, Yuantao Zhou, Zhanling Liu, Haishun Wang, Yaqi Zhao, Rongrong Li, Youpeng Lai, Shujing Gao

**Affiliations:** 1 College of Agriculture and Animal Husbandry, Qinghai University, Xining 810016, China; kgx3272647933@163.com (G.K.); zhouyt@qhu.edu.cn (Y.Z.); liu20000607ling@163.com (Z.L.); 15500741771@163.com (H.W.); 15319205805@163.com (Y.Z.); l2198669810@163.com (R.L.); 2 Qinghai Provincial Key Laboratory of Integrated Pest Management in Agriculture, Qinghai University, Xining 810016, China; 3 Institute of Plant Protection, Qinghai Academy of Agricultural and Forestry Sciences, Qinghai University, Xining 810016, China; 4 Grassland Research Institute, Chinese Academy of Agricultural Sciences, Hohhot 010010, China

**Keywords:** *Gynaephora qinghaiensis*, wing development, *GqBurs*(α), functional studies

## Abstract

The tanning hormone (Bursicon) is a heterodimer secreted by the insect nervous system, playing a crucial role in the process of cuticle darkening and hardening and wing expansion in insects. In this study, we knocked out the *GqBurs* gene using RNAi, thereby impairing the flight ability of adult males by affecting wing development, laying the groundwork for further control of the damage they cause to alpine meadows. The *GqBurs* gene was successfully characterized and expressed at all developmental stages of *Gynaephora qinghaiensis*, with higher expression in 5th instar larvae and wing primordia. In order to investigate the potential role of the *Burs* gene in wing development, we used RNAi technology to inhibit the expression of the *GqBurs* gene by in vivo injection of *dsGqBurs* into male adults of *G. qinghaiensis*, with the aim of affecting their flight ability. The silencing efficiency of the *GqBurs* gene was 86.12%, 97.27%, 65.61%, and 30.51% in each tissue (thorax, abdomen, wing, and wing base) 12 h after *dsGqBurs* injection. In addition, 12 h after injection of exogenous hormones (20E, JH, and Insulin) and a PKA inhibitor (H-89), we found that the *GqBurs* gene was affected by 20E, H-89, JH, and Insulin, and its overexpression and gene silencing were observed in the thorax, abdomen, wings, and wing primordia of male adults of *G. qinghaiensis*. These results indicate that the *GqBurs* gene plays a crucial role in the wing development of male adults of *G. qinghaiensis*. Furthermore, a decrease in *GqBurs* gene expression further affects the extension and expansion of the wings, thereby impacting the insect’s flight ability.

## 1. Introduction

*Gynaephora qinghaiensis* Chou & Ying, 1979 [1] is one of the most severe pests affecting the alpine meadow grasslands of the Qinghai Tibet Plateau. *G. qinghaiensis* feeds on *Festuca rubra* L. and other grassland vegetation. When infestations occur, the pest population density is high, the affected area is extensive, and the damage is severe [2]. Moreover, *G. qinghaiensis* exhibits strong reproductive capacity and demonstrates remarkable adaptability to extreme environments, such as intense ultraviolet radiation, oxygen deprivation, and severe cold, making its control particularly challenging [3]. Under these adverse conditions, the economic development of highland grazing areas has been negatively impacted. More significantly, the persistent damage caused by pests to grassland vegetation has led to changes in plant community structure, further exacerbating the deterioration of grassland ecosystems [4,5]. The grassland ecosystems of the Qinghai Tibet Plateau region are extremely fragile and difficult to restore once damaged. Therefore, implementing effective pest control measures in this area is of paramount importance [4].

The development of insect wings confers significant advantages in migration, foraging, evading predators, mating and reproduction, and withstanding adverse environmental conditions. Research on wings holds substantial importance across multiple fields, including evolutionary biology, ecology, and physiology. The normal progression of wing development is subject to precise regulation by numerous genes and signaling pathways [6,7,8]. The wing and leg primordia of *Drosophila* (Diptera: Drosophilidae) both originate from the epidermis of the fifth abdominal segment. During early embryonic development, the wing disc contains only about 10 cells, and the wing and leg primordia are not yet fully separated. The precise location of the wing primordium is also undetermined [9,10]. During its formation, dorsal cells secrete the Decapentaplegic (Dpp) protein, whose concentration decreases gradually from dorsal to ventral [9,10]. In the region near dorsal cells, the *Distal-less* (*Dll*) gene expresses Vestigial (*Vg*) under stimulation by high concentrations of *Dpp*. These cells containing the Vg protein gradually migrate toward the dorsal side, forming wing primordia. Meanwhile, under the influence of low concentrations of *Dpp* at the distal end, *Escargot* is expressed, leading to the formation of leg primordia. Thus, by late embryonic development, wing and leg primordia are completely separated, with wing disc cell numbers proliferating to approximately 5000 [9,10,11]. Key genes in the wing development signaling pathway can also participate in and regulate insect wingspan.

The physiological processes of insects, including growth and development, metabolism, and behavior, are coordinated and regulated by multiple hormones, such as ecdysone, juvenile hormone, and tannin hormone. Bursicon, a heterodimeric protein that regulates immune function by modulating the insect immune system, controls post-eclosion cuticle darkening and hardening while promoting wing extension and maturation. By binding to the receptor LGR2, it activates the cAMP signaling pathway, thereby regulating insect wing formation and cuticle tanning [12,13,14,15]. RNAi experiments on *Plutella xylostella* (Lepidoptera: Plutellidae) have shown that suppression of normal *Bursicon* gene expression leads to developmental delay and even death and have confirmed that the *Bursicon* gene regulates cuticular pigmentation in insects [16]. Following silencing of the *BmBursicon* gene in *Bombyx mori* (Lepidoptera: Bombycidae), transcriptional levels of wing unfolding-related pathways and genes were examined. Results reveal significant downregulation of wing development-associated genes in both the Wnt and Hippo pathways, accompanied by a wing-wrinkling phenotype [17,18]. Research utilizing the UAS-GAL4 double-hybrid system to investigate the function of the *Drosophila Bursicon* gene revealed that following mutation of the *Bursicon* gene, the fruit fly’s wings cannot extend normally [19,20]. Lu et al. employed RNAi technology to silence *AcBursicon-α* and *AcBursicon-β* in *Aphis citricidus* (Hemiptera: Citricidus), inducing wing deformities in aphids [21]. Furthermore, feeding with the protein kinase A inhibitor (H-89) increased the proportion of deformed wings. Co-feeding dsRNA (*AcBurs-α* and *AcBurs-β*) with H-89 significantly downregulated the wing development-related genes *Nubbin*, *Vestial*, *Notch*, and *Spalt major* [21]. The *Bursicon* gene identified in this study may be associated with wing development in *G. qinghaiensis*, and its functional role in wing development warrants further investigation.

In order to clarify the relationship between tanning hormone proteins and genes related to the wing development of male adults of *G. qinghaiensis* and their roles in the wing development of *G. qinghaiensis*, this paper analyzed the function of tanning hormone proteins in *G. qinghaiensis* by using RNAi technology. The expression level of *GqBurs* was reduced by synthesizing *dsGqBurs* in vitro and injecting *dsGqBurs* into male adults of *G. qinghaiensis*. Using qRT-PCR, we examined the expression of the *GqBurs* gene at different developmental stages (5th–7th instar larvae and pupal stage) and different tissues (thorax, abdomen, wings, and wing primordia) of adult males of *G. qinghaiensis*, and we explored the effects of three exogenous hormones (20-hydroxyecdysone, Juvenile hormone, and Insulin) and a PKA inhibitor (H-89) on the *GqBurs* gene. In conclusion, our study reveals the functional role of tanning hormone proteins on the wing development of *G. qinghaiensis* and provides theoretical and data support that can support future research in this field.

## 2. Results

### 2.1. Phylogenetic Analysis of the GqBurs Gene of Gynaephora qinghaiensis

**Figure 1:**
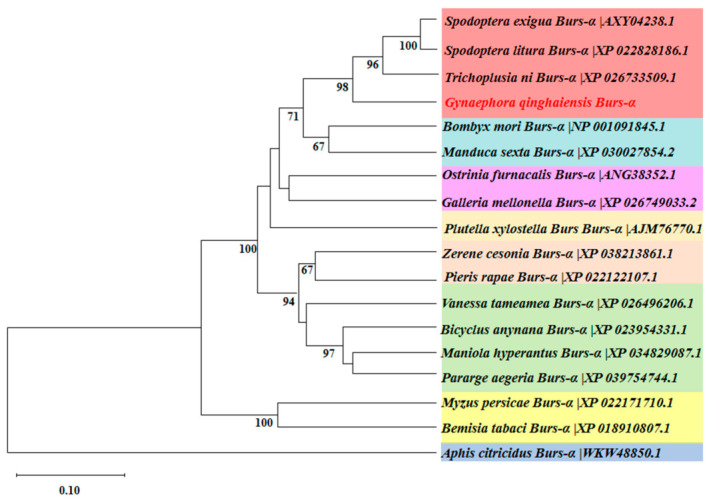
Phylogenetic analysis of the *Bursicon* genes in 18 insect species. Note: *Spodoptera exigua* Burs (α) (AXY04238.1); *Spodoptera litura Burs* (α) (XP_022828186.1); *Trichoplusia ni* Burs (α) (XP_026733509.1); *Bombyx mori* Burs (α) (NP_001091845.1); *Manduca sexta* Burs (α) (XP_030027854.2); *Ostrinia furnacalis* Burs (α) (ANG38352.1); *Galleria mellonella* Burs (α) (XP_026749033.2); *Plutella xylostella Burs* (α) (AJM76770.1); *Zerene cesonia* Burs (α) (XP_038213861.1); *Pieris rapae* Burs (α) (XP_022122107.1); *Vanessa tameamea* Burs (α) (XP_026496206.1); *Bicyclus anynana* Burs (α) (XP_023954331.1); *Maniola hyperantus* Burs (α) (XP_034829087.1); *Pararge aegeria* Burs(α) (XP_039754744.1); *Myzus persicae* Burs (α) (XP_022171710.1); *Bemisia tabaci* Burs (α) (XP_018910807.1); *Aphis citricidus* Burs (α) (WKW48850.1).

In this study, the open reading frames (ORFs) of *GqBurs* were 480 bp in length. These ORFs encode polypeptides consisting of 159 amino acid residues. Based on sequence similarity, this gene was designated as *GqBurs* (α). The amino acid sequence of *GqBurs* from *Gynaephora qinghaiensis* was aligned with that of 17 other insect species using the blast program from NCBI, and a phylogenetic tree was constructed to analyze its phylogenetic relationships. Phylogenetic analysis results show that these 17 insect species are clearly divided into two branches. *GqBurs* and *AcBurs* (outgroup) are clearly clustered into their respective branches. *GqBurs* exhibits the highest genetic similarity with *TnBurs*, with a confidence level of 98, followed by *SlBurs* and *SeBurs*. This suggests that the sequence of *GqBurs* may share homology with the sequences of these insects (Figure 1).

### 2.2. The Developmental Expression Profile of the GqBurs Gene of Gynaephora qinghaiensis

**Figure 2:**
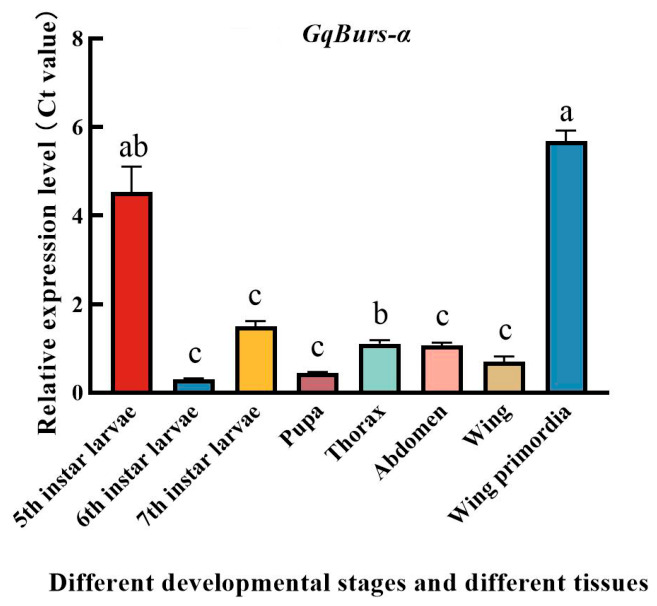
Analysis of the expression profile of the *GqBurs* gene in *Gynaephora qinghaiensis* at different developmental stages and in different tissues. N5–N7: 5th–7th instar larvae; all means (mean ± standard error) are based on 3 biological replicates. Significant differences between stages are indicated by lowercase letters above each bar (one-way ANOVA followed by Tukey’s test; *p* < 0.05).

RT-qPCR was performed to detect the expression level of messenger RNA of the *GqBurs* gene, which was expressed at all developmental stages. The expression was stable in 7th instar larvae, the thorax, the abdomen and mid-wing (male adults); highest in 5th instar larvae and at the base of the wings (male adults); and lowest in 6th instar larvae and at the pupal stage (male pupae). The expression of the *GqBurs* gene in 5th instar larvae was 15 and 3.2 times higher than in 6th and 7th instar larvae, respectively. The expression of the *GqBurs* gene in wing primordia was 5.6 times higher than that in both the thorax and abdomen, and 9.3 times higher than that in wings. The expression of the *GqBurs* gene in the body and wing primordia of 5th instar larvae was 11.25 and 14 times higher than that at the pupal stage, respectively (Figure 2).

### 2.3. The Effects of Burs Gene Suppression on Wing Size in Male Adults and Burs Expression Levels

**Figure 3:**
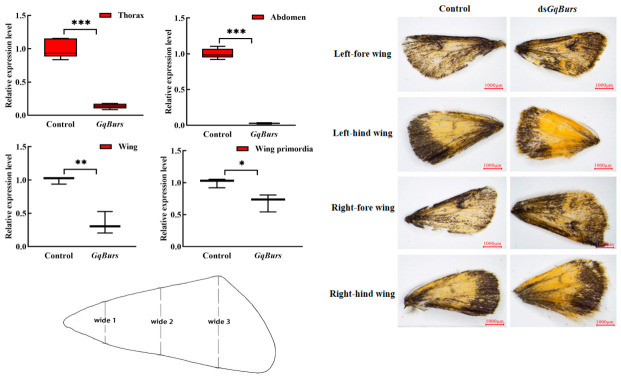
Analysis of the gene expression profile of the *GqBurs* gene and wing phenotypes in *Gynaephora qinghaiensis* following RNAi interference. Note: Wide 1-Wide 3: 3 widths measured from base to edge. The left figure shows the expression patterns of the *GqBurs* gene in the thorax, abdomen, wings, and wing primordia following injection of ds*GqBurs*. The image on the right shows the wing characteristics observed after injection of ds*GqBurs*. All of the means (±standard error of the mean) are based on 3 biological replicates. The significant difference between the treatment and control groups is indicated by asterisks (Student’s *t*-test). ns, no significance; * *p* < 0.05; ** *p* < 0.01; *** *p* < 0.001.

RNAi technology was used to study the biological function of the *GqBurs* gene in the wing development of male adults of *G. qinghaiensis*. A total of 12 h after injection, the silencing efficiency in the wing base was lower than that in other tissues (*p* < 0.05). Their anterior and posterior wing lengths on the left and right sides were reduced at a significant level (*p* < 0.05) compared to the control (Figure 3). The widths of Wide 1 and Wide 2 on the right-hind wing showed no significant difference compared with the control group, with values of 626.15 ± 3.15 µm and 2605.78 ± 5.41 µm respectively. The width of Wide 3 on both the left and right sides of the fore and hind wings was significantly reduced compared with the control group (*p* < 0.05), with values of 3066.55 ± 9.82 µm, 2750.52 ± 5.2 µm, 3159.56 ± 6.86 µm and 3134.31 ± 6.1 µm respectively (Table 1). That the length and width of the wings of male adults from *G. qinghaiensis* decreased after injection of *dsGqBurs* indicates that RNAi interference effectively interfered with the *GqBurs* gene of *G. qinghaiensis* and also suggests that the *GqBurs* gene plays an important role in the wing development of insects (Table 1).

### 2.4. The Effects of Injection of 20E, JH, Insulin and H-89 on the Expression of Bur in Male Adults

**Figure 4:**
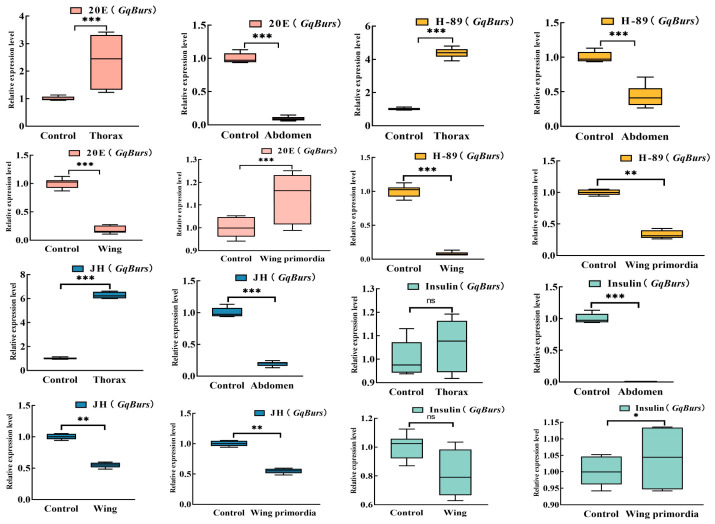
Expression profiles of *GqBurs* genes under treatments of three exogenous hormones and a PKA inhibitor. Note: All of the means (±standard error of the mean) are based on 3 biological replicates. The significant difference between treatment and control is indicated by asterisks (Student’s *t*-test). ns, no significance; * *p* < 0.05; ** *p* < 0.01; *** *p* < 0.001.

20E, H-89, JH, and Insulin were injected into the intersegmental membrane between the 2nd and 3rd pairs of thoracic peduncles in the thorax of 1-day-old male adults of *G. qinghaiensis*, respectively, using a microinjector. A total of 12 h after injection of these three exogenous hormones and a PKA inhibitor, we found that the *GqBurs* gene was affected by 20E, H-89, JH, and Insulin, and its overexpression and gene silencing were observed in the thorax, abdomen, wings, and wing primordia of male adults of *G. qinghaiensis*. After 20E injection, the *GqBurs* gene showed highly significant expression levels in the thorax and wing primordia (overexpression efficiency of 135.74% and 13.51%, respectively) and decreased expression levels in the abdomen and wings (silencing efficiency of 90.58% and 81.91%, respectively) (*p* < 0.05). After injection of H-89, the *GqBurs* gene showed highly significant expression levels in the thorax (overexpression efficiency of 337.10%, respectively) and decreased expression levels in the abdomen, wings and wing primordia (silencing efficiencies of 56.52%, 92.10% and 66.84%, respectively) (*p* < 0.05). After injection of JH, the *GqBurs* gene showed highly significant expression levels in the thorax (overexpression efficiency of 534.91%, respectively) and decreased expression levels in the abdomen, wing and wing base (silencing efficiency of 80.53%, 45.20%, 45.20%, respectively) (*p* < 0.05). After Insulin injection, the *GqBurs* gene showed highly significant expression levels in the thorax and wing primordia (overexpression efficiency of 5.61% and 4.07%, respectively) and decreased expression levels in the abdomen and wings (silencing efficiency of 99.86% and 18.77%, respectively) (*p* < 0.05). Results were consistent after injections of 20E and insulin and after injections of H-89 and JH (Figure 4).

## 3. Discussion

In our study, we found the presence of tanning hormone protein genes in *Gynaephora qinghaiensis*. In this study, we silenced the *GqBurs* gene by the RNAi method to lay the foundation for further control of its damage to alpine meadow grasslands and to provide data support for subsequent control. In addition, the *GqBurs* gene clusters on the phylogenetic tree with homologs of several lepidopteran insects, and these results suggest compliance with the nomenclature rules of the *G. qinghaiensis GqBurs* gene.

RT-qPCR examination of messenger RNA expression levels of the *GqBurs* gene revealed relatively stable expression in 5th instar larvae and adult males (thorax, abdomen, and wings). It has been shown that *AcBurs-α* and *AcBurs-β* are highly expressed in fourth instar winged nymph and adult winged *Aphis citricidus* and are closely associated with wing development in *Aphis citricidus* [21]. High expression levels of the *Burs* gene at the pupal stage of *Anopheles gambiae* compared to larvae and adults of *Aphis citricidus* suggest that Burs may play a role in pupal development and metamorphosis [22]. In our study, the expression of the *Bursicon* gene was highest in 5th instar and 7th instar larvae and in the wing primordia of adult males of *G. qinghaiensis* and lowest in 6th-instar larvae and during the pupal stage (male pupae). We speculate that this may be because *G. qinghaiensis* enters a period of voracious feeding during this stage, absorbing large amounts of nutrients, which leads to changes in hormone levels and consequently affects the expression of the *GqBurs* gene. Therefore, it is hypothesized that this gene plays an important role in the development and metamorphosis process of the larval and pupal stages of *G. qinghaiensis*, as well as in the wing extension process of the adult stage. Inhibition of *Burs* gene expression was found to be lethal to pre-adult *Drosophila*, thus preventing normal fledging [23]. Similarly, feeding *dsBurs* to *Plutella xylostella* late 4th instar larvae resulted in developmental stagnation of *Plutella xylostella* at the pupal stage where it was unable to fledge and eventually died [16]. It has also been shown that Hippo induces wing growth by activating the transcription of the Yorkie gene, which promotes cell proliferation and survival and inhibits apoptosis, and that when the activity of Yorkie is lost, no wings can grow [24]. The Hippo signaling pathway activates Vestigial and Wingless, which initiates the wing growth and development process [25]. After the injection of *dsBurs* in *Bombyx mori*, the wing spreading process of adults could not be completed normally, and their egg laying was also reduced, which affected the expression of Vitellogenin and Vitellogenin receptor genes and the expression of Fat of the Hippo signaling pathway and the *Dachsous* gene, and the expression of Fat, Dachsous and Wingless of the Hippo signaling pathway was significantly downregulated after the suppression of *Burs* gene expression, suggesting that the *Burs* gene may regulate the expression of Wingless, which in turn affects the transcription of Dachsous and Yorkie, resulting in the failure of the wing extension of *Bombyx mori* [26]. The *Burs* gene not only affects the development of insect wings but can also affect the growth and development of the next generation of *Tribolium castaneum* (Polyphaga: Tenebrionidae), which affects adult egg production and hatchability through the regulation of Vg and VgR. Adults laying eggs after disrupting the expression of *Burs-α* have a smaller egg size, and dissections of unhatched eggs from the *dsBurs* treatment group reveal that larval development is severely impaired. The development of larvae in the *dsBurs*-treated group was severely impeded [27]. The *GqBurs* gene exhibits the lowest silencing efficiency in the wing primordia. Silencing the *Burs* gene severely affects the flight ability of *G. qinghaiensis* wings. We speculate that silencing *GqBurs* indirectly affects the expression level of the Yorkie gene, thereby inhibiting cell proliferation and survival and accelerating cell apoptosis. This leads to the contraction of the normal wingspan, which in turn affects the normal flight ability of male adults of *G. qinghaiensis*, turning their advantages in migration, foraging, and courtship into disadvantages. The results of our speculation align perfectly with the objectives of our study, thereby providing important theoretical support for the control of grasshoppers in alpine meadow ecosystems and identifying key targets for maintaining the ecological balance of these grasslands. In our results, we also found that the length and width of the anterior and posterior wings of the right and left wings of male adult *G. qinghaiensis* were significantly reduced after *dsGqBurs* injection compared with the control group (*p* < 0.05), which confirmed what we had hypothesized earlier.

Three hormones (20E, JH and Insulin) have been widely reported to be involved in aphid wing development [27,28,29,30,31]. Hormone treatment of *Bombyx mori* revealed that treatment of *Bombyx mori* larvae back with JH or the JH analog methoprene resulted in extra molting of the larvae and the production of over-aged larvae and also inhibited the growth differentiation of the wing primordia, whereas 20E promoted the growth differentiation of the wing primordia, and a similar situation was observed in *Tribolium castaneum* [32,33]. Deng et al. found that the wing primordial epidermal protein gene BmWCP4, which is mainly expressed in the epidermis of prepupae and mid-pupae, is a key factor in the growth and differentiation of the wing primordium, and that 20E activated the expression of BmWCP4 by inducing the expression of the transcription factors BmPOUM2 and BmFTZ-F1, whereas JH inhibited this process [34,35]. During pupal development, the juvenile-preserving hormone prevents the developmental differentiation of certain organ buds in adult insects, which can form permanent pupae or pupa–adult intermediates and die [36]. The lifespan of adult females of *Drosophila* and *Helicoverpa armigera* (Lepidoptera: Noctuidae) is reduced by treatment with a juvenile-preserving-hormone analogue [37]. Thus, the regulation of wing primordium development by JH and 20E proceeds through a complex and systematic regulatory network. Lu et al. showed that there was no significant change in *AcBurs* gene expression levels after feeding 20E, JHIII and Insulin to 4th instar *Aphis citricidus* nymphs, suggesting that the regulation of wing development in adult *Aphis citricidus* by the *AcBurs* gene does not depend on these three hormones, but the feeding of a PKA inhibitor caused *Aphis citricidus* wing deformities after feathering [21]. The *Burs* gene has been reported to regulate insect wing development by inducing apoptosis in wing dermal cells through activation of the cAMP/PKA signaling pathway [22,38]. Because wing expansion during adult molt is specifically regulated by *Burs* genes and crustacean cardioactive peptide (CCAP), *Burs* genes are also involved in the mechanism of post-molt cuticle tanning and wing extension [38,39]. In our study, after injection of 20E and Insulin into male adults of *G. qinghaiensi*, the *Burs* gene showed highly significant expression levels (*p* < 0.05) in the thorax and wing primordia and decreased expression levels in the abdomen and wings, which was hypothesized to be possibly due to the fact that high-quality concentration of 20E promotes the development of the wing primordium and facilitates the completion of the metamorphosis process. After injection of H-89 and JH, the *Burs* gene showed highly significant expression levels in the thorax (*p* < 0.05) and decreased expression levels in the abdomen, wing and wing primordia, which was hypothesized to be possibly due to the inhibition of the cAMP/PKA signaling pathway by a high mass concentration of H-89, inducing apoptosis of wing dermal cells and inhibition of growth and differentiation of wing primordial bases by JH, which led to the decreased expression levels.

## 4. Materials and Methods

### 4.1. Rearing and Sample Preparation of Experimental Insects

The larvae of *Gynaephora qinghaiensis* used in this experiment were collected from Haiyan County, Haibei Prefecture, Qinghai Province (36°59′ N, 100°52′ E, elevation 3095 m), and placed in insect-rearing cages (50 cm × 50 cm × 50 cm, with no substrate present in the cages). They were fed with *Festuca rubra* L., collected from the wild, in the laboratory Rearing conditions: temperature, (26 ± 1)°C; relative humidity, 60%~80%; photoperiod, L/D = 16 h/8 h] until they emerged as adult males and females. Samples were collected at different developmental stages of *G. qinghaiensis* to study the relative expression levels of *GqBurs*. The samples consisted of 5th instar larvae (N5), 6th instar larvae (N6), 7th instar larvae (N7), and adult males [their thorax, abdomen, wings, and wing primordia were dissected for reserve]. All adults were 1 d old adults. There were three biological replicates for each treatment, three *G. qinghaiensis* larvae per biological replicate for 5th–7th instars (N5–N7), and 10 male adults per biological replicate for male adults. Immediately after collection, all samples were immersed in TRIzol reagent and stored at −80 °C to prevent RNA degradation for subsequent extraction.

### 4.2. Phylogenetic Analysis

*GqBurs* was identified based on the *G. qinghaiensis* genome database. Sequence homology searches were performed using NCBI’s BLASTn program (http://www.ncbi.nlm.nih.gov/BLAST/, accessed on 20 April 2025), and the amino acid sequences of tanning hormones from 17 Lepidoptera species with high similarity were selected. Multiple sequence alignment was performed using ClustalW in the BioEdit software (v7.0.9.0). Based on the sequence alignment results, a phylogenetic tree was constructed using the Neighbor-Joining (N-J) method in the MEGA software (v10.1.7), with each branch tested 1000 times.

### 4.3. RNA Extraction and qRT-PCR

Total RNA was extracted from each sample (4 tissues) using the Trizol method. The purity of RNA from each tissue was assessed individually using a spectrophotometer. Samples with RNA purity within the acceptable range (OD260 nm/OD280 nm of 1.8-2.2) were converted to cDNA using a reverse transcription kit (M-MLV) (Beijing SolarBio Science, Beijing, China). The resulting cDNA was stored at −20 °C for future use. Using synthetic cDNA samples (from the thorax, abdomen, wings, and wing primordia of male adults) as templates, each of the four samples contained three biological replicates, with three technical replicates performed for each biological replicate. Amplification reaction (20 μL): a total of 0.8 μL of forward primer, 0.8 μL of reverse primer, 10 μL of Master Mix, 7.4 μL of ddH_2_O, and 1 μL of cDNA template. Reactions were performed on a real-time quantitative PCR instrument (ABI7500 model). PCR amplification conditions: pre-denaturation at 95 °C for 1 min, 95 °C denaturation for 15 s, annealing (at 60 °C) for 15 s, 72 °C for 45 s, and repeated for 40 cycles. Rps15 was used as an internal reference gene to calculate the relative expression of *GqBurs* genes. Quantitative primers were designed using Prime 5.0 (Table 2).

### 4.4. RNAi

Based on the identified gene *GqBurs*, an RNA interference primer was synthesized using Prime Blast, with the 5′ end containing the T7 promoter sequence (TAATACGACTCACTATAGGG). Using cDNA from different tissues of *G. qinghaiensis* as templates, gene sequences containing the T7 promoter were amplified via RT-PCR. PCR amplification was performed, followed by purification of PCR products using Omega (Omega Bio-Tek, Norcross, GA, USA) purification reagents. The purified amplified products were ligated into the PGEM-TEasy vector (Promega, Madison, WI, USA), then subjected to bacterial culture PCR verification and sequencing. Plasmid extraction was carried out using the TIANGEN Plasmid Extraction Kit. Finally, dsRNA interference fragments were transcribed and synthesized using Promega’s T7RiboMAXTM Express RNAi System. In the experimental group, 1 µL (1000 ng/µL) of dsRNA (dsBurs) was injected using a microinjector into the inter-segmental membrane between the second and third thoracic legs of the thorax of 1-day-old male *G. qinghaiensis* moth adults. The dsGreen fluorescent protein was used as the control group.

### 4.5. The Effects of Hormone and PKA Inhibitor Injections on Gene Expression Levels During the Adult Stage

A total of 1 μL of 20E (0.5 μg/μL) [29], H-89 (1.25 mg/mL) [21], JH (1 μg/mL) [28], and Insulin (5 mg/mL) [30] were injected into the inter-segmental membrane between the second and third thoracic legs of the thorax of 1-day-old male *G. qinghaiensi*. Using ddH_2_O as the control, samples were frozen after 12 h (10 individuals per replicate, with 3 replicates). Photographs of the wing surfaces of hormone-injected males were taken for subsequent measurements of wing circumference and area. RNA extraction and RT-qPCR procedures were identical to those in Section 4.3.

### 4.6. Data Processing

All data are expressed as means ± the standard error of 3 independent biological replicates. The relative expression of *GqBurs* in different tissues was calculated by the 2^−ΔΔCT^ value method using the expression in the thorax of male adults as a control. The relative expression of the *GqBurs* gene in different tissues after RNAi interference was calculated by the 2^−ΔΔCT^ value method using the expression of dsGFP as a control. One-way analysis of variance (ANOVA) was performed using SPSS v.25.0 for Windows (IBM, Armonk, NY, USA) (*p* < 0.05). And differences in *GqBurs* expression in different tissues and under different treatments were tested by a t-test using the Graphpad prism software (9.5) (* *p* < 0.05; ** *p* < 0.01; ns, not significantly different), and tissue expression profiles were plotted.

## 5. Conclusions

In summary, we identified the *GqBurs* gene in *Gynaephora qinghaiensis* and artificially injected in vitro synthesized dsRNAs (ds*GqBurs*) into 1-day-old male adults to interfere with the expression of the *GqBurs* gene. The results show that the *GqBurs* gene was disturbed, and its expression was significantly reduced, with a corresponding reduction in the length and width of its wings and restricted development. Therefore, the tanning hormone encoded by the gene for this protein may participate in the regulation of insect wing development and cuticle tanning [38,39]. Therefore, the tanning hormone encoded by the gene for this protein may participate in regulating insect wing development, immunity, and cuticle tanning. Sprayable double-stranded RNA formulations or genetically modified control methods could be developed, providing feasible measures for pest management. The *Burs* gene was spatiotemporally and temporally expressed in larval, pupal and adult wing development, and silencing of the gene significantly inhibited adult wing development and flight ability, resulting in death, thus verifying its key role in regulating insect survival and wing function and providing a basis for targeted regulation for insect pest control in alpine meadows. 20E, Insulin, JH and PKA inhibitors (H-89) differentially regulate the expression of this gene: 20E and Insulin promote the expression of thoracic and wing basal genes, while JH and PKA (H-89) inhibitors reduce the expression level by inhibiting wing primordial differentiation and apoptosis, which will reveal the complex regulatory network mechanism of hormone-gene interactions and provide a basis for the development of an ecological prevention and control strategy based on the gene-hormone pathway. This will provide a scientific basis for the development of ecological control strategies based on gene-hormone pathways. This suggests that the *GqBurs* gene plays an indispensable role in the wing expansion of male adults of *G. qinghaiensi*.

## Figures and Tables

**Table 1:** Changes in wing length after RNAi disturbance.

Treatment	Fore-Hind Wing	Length (µm)	Wide 1 (µm)	Wide 2 (µm)	Wide 3 (µm)
Control	Left-fore wing	5782.5 ± 8.51 a	597.0 ± 1.23 a	2764.3 ± 22.26 a	3442.3 ± 9.45 a
	Left-hind wing	5612.4 ± 2.14 a	537.7 ± 4.55 a	2730.5 ± 42.82 a	3093.0 ± 5.04 a
	Right-fore wing	5550.6 ± 9.40 a	538.1 ± 7.10 a	2374.3 ± 22.23 a	3274.8 ± 4.39 a
	Right-hind wing	5588.4 ± 4.33 a	640.8 ± 6.61 a	2663.0 ± 7.32 a	3522.0 ± 5.03 a
*GqBurs*	Left-fore wing	5287.5 ± 6.81 b	482.2 ± 7.10 b	2281.9 ± 8.5 b	3066.5 ± 9.82 b
	Left-hind wing	5034.4 ± 8.48 b	336.9 ± 8.80 b	2318.4 ± 5.95 b	2750.5 ± 5.20 b
	Right-fore wing	5122.7 ± 8.04 b	491.6 ± 6.19 b	2120.6 ± 80 b	3159.5 ± 6.86 b
	Right-hind wing	5042.0 ± 5.90 b	626.1 ± 3.15 a	2605.7 ± 5.41 a	3134.3 ± 6.10 b

Note: A total of 30 insects were measured, with 10 insects constituting one replicate. This method effectively reduces data errors. Wing length refers to the distance from the wing base to the wing tip (typically measured along the central wing). Wing width measurements were taken as illustrated below (this study measured three widths: Width 1 at the wing’s base, Width 2 at the wing’s midpoint, and Width 3 at the wing’s apex). Stereo microscopy was used to photograph the wings, and the Image J (1.x) software was employed to measure the required wing length and width data. Changes in wing length and width following ds*GqBurs* injection, with dsGFP as the control. In Table 1, lowercase letters indicate significant differences in wing length and width between the treatment group and the control group (after one-way ANOVA followed by Tukey’s test; *p* < 0.05).

**Table 2:** Primers used in the study.

Primers	Primer Sequence	Use
T7 promoter	TAATACGACTCACTATAGGG	-
*GqBurs*	F: T7 + GGAAGTGCCTTTGTCACCTGG	dsRNA synthesis primer
	R: T7 + GGAGCTTTCGTAGTGATCTTTCTG	
*GqGFP*	F: T7 + CACAAGTTCAGCGTGTCCG	dsRNA synthesis primer
	R: T7 + TTCACCTTGATGCCGTTC	
*GqBurs*	F: TTGCCAAGAATCTGGTGAG	qRT-PCR
	R: TCCTCAATACCGCCACAT	

## Data Availability

The original contributions presented in the study are included in the article, and further inquiries can be directed to the corresponding author.
